# A Validated RP HPLC-PAD Method for the Determination of Hederacoside C in Ivy-Thyme Cough Syrup

**DOI:** 10.1155/2010/478143

**Published:** 2010-08-24

**Authors:** Ayman Khdair, Mohammad K. Mohammad, Khaled Tawaha, Eman Al-Hamarsheh, Hatim S. AlKhatib, Bashar Al-khalidi, Yasser Bustanji, Samer Najjar, Mohammad Hudaib

**Affiliations:** ^1^Faculty of Pharmacy, Applied Sciences University, Amman 11931, Jordan; ^2^Department of Pharmaceutical Sciences, Faculty of Pharmacy, University of Jordan (UJ), Amman 11942, Jordan; ^3^Department of Pharmaceutics and Pharmaceutical Technology, Faculty of Pharmacy, University of Jordan (UJ), Amman 11942, Jordan; ^4^Department of Clinical Pharmacy and Biopharmaceutics, Faculty of Pharmacy, University of Jordan (UJ), Amman 11942, Jordan; ^5^Sana Pharmaceutical Research Co., Amman 11931, Jordan

## Abstract

A simple reversed phase high-performance liquid chromatographic (RP-HPLC) method coupled with a photodiode array detector (PAD) has been developed and validated for the analysis of hederacoside C, the marker of ivy plant, in Ivy-Thyme cough syrup. Separation of hederacoside C was achieved using a Phenomenex-Gemini C18 column isothermally at 40°C. A mobile phase system constituted of solvent A (water: acetonitrile: orthophosphoric acid (85%), 860 : 140 : 2 v/v) and solvent B (acetonitrile: orthophosphoric acid (85%), 998 : 2 v/v) was used, at gradient conditions, at a flow rate of 1.5 mL/min. Analysis was performed using UV-detection (205 nm). The method was linear over the range (0.03–0.15) mg/mL of hederacoside C (*r* = 0.9992). Repeatability and intermediate precision were acceptable (RSD <2%). Limits of detection (LOD) and quantitation (LOQ) were 0.011 and 0.032 mg/mL, respectively. Percentage recovery was found to lie between 99.69% and 100.90% (RSD <2%). The method was also proved to be specific (peak-purity coefficient = 0.996).

## 1. Introduction

In recent years there has been a growing interest in the therapeutic use of herbal medicines or phytopharmaceutical products [1, 2]. The reasons of such interest can be referred mainly to the fears of abuse and misuse of synthetic drugs, the side effects or inefficacy of conventional medicine, the access to conventional pharmacological treatment is not available to whole population, and the suggestions regarding the safety of natural products [3]. 

**Figure 1:**
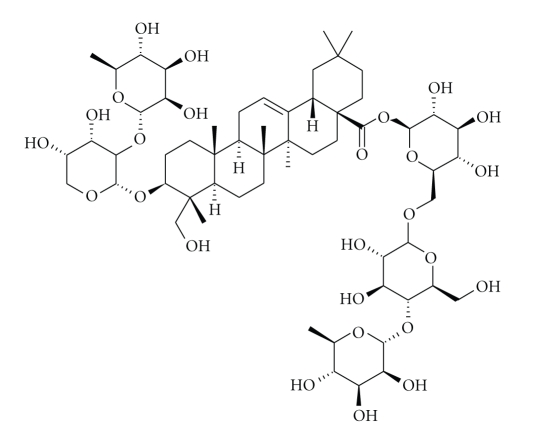
Chemical structure of hederacoside C (3-[[2-O-(*α*-L-Rhamnopyranosyl)-*α*-L-arabinopyranosyl]oxy]-23-hydroxyolean-12-en-28-oic acid 6-O-[4-O-(*α*-L-rhamnopyranosyl)-*β*-D-glucopyranosyl]-*β*-D-glucopyranosyl ester).

Ivy-Thyme cough syrup is an herbal medicine that combines the extract of *Hedera helix* (English ivy, family: Lamiaceae) leaves along with the extract of *Thymus vulgaris* (thyme, family: Araliaceae) herb for the purpose of cough treatment. As a medicinal plant, *H. helix *leaf extract is approved by the German Commission E [4] for its efficacy against chronic inflammatory bronchial conditions and productive coughs due to its actions as an expectorant and its spasmolytic effect among children and adults [5, 6]. These effects are attributable, in particular, to the therapeutically important constituents of the ivy leaf extract, which belong to the class of triterpene saponins such as hederacoside C (Figure 1), a marker and an active ingredient of ivy leaf extract [7–10]. On the other hand, thyme is a medicinal herb whose leaf extract is approved by commission E in the treatment of bronchitis, whooping cough, and upper respiratory inflammation. Thyme and its various extracts are well known for their bronchospasmolytic, expectorant, and antibacterial effects [11]. These latter effects are most likely attributed to the phenolic components (thymol and carvacrol) of the plant volatile oil [12, 13].

To evaluate the quality of herbal medicines, reliable analytical methods have to be applied to the quantitative determination of the constituents with known therapeutic activity or to the markers in final products [1, 14]. For the quality control of Ivy-Thyme cough syrup, hederacoside C and thymol, the active markers of ivy and thyme [15], respectively, are needed to be quantified by a validated method in order to determine the contents of their corresponding plant extracts in the finished product.

Accordingly, the objective of the present study was to develop a simple, reliable, and validated analytical method for the quantification of hederacoside C, the marker of ivy, by the use of high-performance liquid chromatography (HPLC) with UV detection, a method that can be used efficiently for routine quality control and analysis of such herbal product.

Actually, the challenge in the development of such a method is attributed to the presence of hederacoside C in a complex matrix of the cough syrup. It seems that the presence of other saponins from other extracts (e.g. thyme extract) used in cough preparations as well as other ingredients of the cough syrup may interfere with the chromatographic peak of hederacoside C and, thus, its quantification. These factors increase the complexity of analysis and necessitate the development of a new analytical method for the accurate quantification of hederacoside C, the quality marker of ivy.

## 2. Experimental

### 2.1. Instrumentation

The HPLC system used was a Shimadzu CLASS-VP System equipped with a model series LC-10 ADVP Pump, FCV-10ALVP Low pressure gradient flow controller valve, SCL-10 ADVP controller, 50 *μ*L loop injector, CTO-10As column oven, and a SPD-M10AVP diode array detector. Data acquisition was performed on class-VP software.

### 2.2. Materials and Reagents

The standardized raw material of *Hedera helix* leaves (standardized to contain 14.8% of hederacoside C) was supplied by Fenzilberg (Germany), hederacoside C standard with purity of more than 99% was purchased from Phytoplan (Germany), orthophosphoric acid (H_3_PO_4_) 85% from Merck (Germany), filter membranes were purchased from Vivid (PTFE/0.45 *μ*m/25 mm diameter), Ivy-Thyme cough syrup (potency: 0.7 mg hederacoside C and 0.2 mg thymol per 1 mL syrup), and placebo were generously provided by Sana Pharmaceutical Research Co. (Jordan). All solvents used were of HPLC grade; acetonitrile and methanol gradient grade were purchased from Scharlau (Spain), bidistilled water was produced via a Bi-Dest 2302 GFL (Germany).

### 2.3. HPLC Conditions

The column was a reversed-phase Phenomenex-Gemini C18 (250 × 4.6 mm i.d.; 5 *μ*m) equipped with a Phenomenex security guard column (4.0 × 3.0 mm i.d.). For HPLC separation, the mobile phase consisted of a binary mixture of solvent-A (H_2_O : ACN : H_3_PO_4_) in the ratio of (860 : 140 : 2) and solvent-B (ACN : H_3_PO_4_) in the ratio of (998 : 2) with a gradient program as follows: 0–60% B (0–40 min), 60–100% B (40–41 min), 100% B isocratic (41–55 min), and return to 0% B (55-56 min), and finally, reconditioning the column with 0% B isocratic (55–70 min). The analysis was performed at a flow rate of 1.5 mL/min. Injection was manual and the injection volume was 50 *μ*L. The UV detector was set at 205 nm and separation was performed isothermally at 40°C. All samples and standards injected are filtered through 0.45 *μ*m membrane filter.

### 2.4. Reference Solution

A reference solution of hederacoside C was prepared at a concentration of 1.0 mg/mL by transferring 10 mg of hederacoside C to a 10 mL volumetric flask, methanol was added, and the solution was sonicated for 10 min then completed to volume.

### 2.5. Standard Solutions

Hederacoside C stock solution containing 1.0 mg/mL of hederacoside C was prepared by transferring 3.378 g of standardized Ivy raw material powder (containing 14.8% hederacoside C) in 500 mL volumetric flask, filled up to 80% of its volume with methanol, then it was sonicated for 30 minutes in the ultrasonic bath to dissolve hederacoside C. After cooling to room temperature, the volume was filled up to the mark with methanol. Calibration standard solutions with different concentrations were prepared by appropriate dilutions from the stock using methanol as the diluent.

### 2.6. Sample Preparation

1.7 g of Ivy-Thyme cough syrup was weighed in 10 mL volumetric flask, filled with 80% of the volume with methanol, sonicated for 15 min to homogenize the solution in ultrasonic bath, and after cooling to room temperature the flask was filled up to the mark. According to this procedure, the final solution is supposed to have a concentration of 0.1 mg/mL hederacoside C.

## 3. Results and Discussion

### 3.1. Method Development

Challenges in the development of an HPLC method with efficient separation of hederacoside C in cough syrup include its separation from a saponin mixture coming from ivy extract along with its separation from other saponins present in the thyme extract. In addition, hederacoside C, like almost all other saponins, lacks a chromophore; it absorbs UV-light at wavelengths below 210 nm [8, 16], which makes its detection in a complex matrix of a herbal product along with its analysis by the use of gradient elution not an easy task [17].

To the best of our knowledge, the present study is the first to report a validated method for the analysis of ivy extract in pharmaceutical products (syrup) and the first on ivy-thyme mixture. In literature, analysis of Ivy plant and extracts, using hederacoside C as a marker, has been generally performed by means of RP-HPLC and UV detection (wavelengths below 210 nm) based mainly on C18 columns under gradient elution [6, 17, 18]. For the analysis of other types of saponins, some of which from other natural sources, comparable methods with modified conditions, solvent systems or detector were also developed and reported [16, 19]. Unfortunately, reproducing these methods to the analysis of Ivy-Thyme syrup under study was always unsuccessful. 

**Figure 2:**
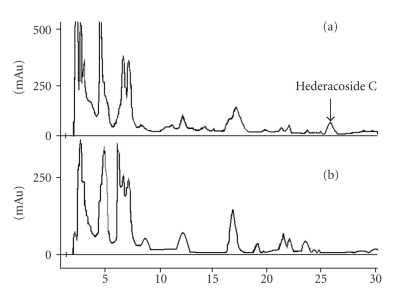
Liquid chromatograms of (a) Ivy-Thyme cough syrup sample and (b) Ivy-Thyme cough syrup placebo.

In the present study, the developed HPLC method ensured sufficient chromatographic separation (Figure 2(a)), and accurate and precise quantification of hederacoside C. Moreover, the use of UV detector, the most common type of detectors used with HPLC instruments [14, 20], makes the developed method a simple and suitable method for routine quality control of ivy-based cough syrups.

### 3.2. Validation of the HPLC Method

The developed method was validated according to the guidelines of the International Conference on Harmonization (ICH) for validation of analytical procedures [21] and USP [22] for its linearity, precision, accuracy, and specificity.

#### 3.2.1. Range

The calibration range over which the analysis should be validated is determined according to the purpose of the analysis. For the quantification of an active ingredient in final product, hederacoside C in cough syrup; the suggested range to be covered according to ICH guidelines is 80%–120% of the test concentration. In our experiment the chosen range was (0.03–0.15) mg/mL of hederacoside C, which corresponds to a range of 30%–150% of the test concentration (0.1 mg/mL).

#### 3.2.2. Linearity

The linearity of the HPLC method for determination of hederacoside C was evaluated by analyzing a series of different concentrations of hederacoside C. According to ICH guidelines, at least five concentrations must be used covering the specified range. In the present experiment, seven different concentrations of hederacoside C were chosen, covering the range of (0.03–0.15) mg/mL of hederacoside C. Each concentration was injected in duplicate. The method was found linear over the specified range with a correlation coefficient (*r*) value of 0.9992 (Table 1).

#### 3.2.3. Precision

For the evaluation of method precision, repeatability and intermediate precision were performed at 100% of the test concentration as guided by ICH. Six samples of the drug, at 0.1 mg/mL concentration level of hederacoside C, were prepared and analyzed by the developed method at the same day for repeatability assessment, and then repeated 24 hours apart for the evaluation of intermediate precision. Coefficient of variation (or relative standard deviation, RSD) has been calculated, the results indicated that the developed method was with acceptable precision (Table 2).

#### 3.2.4. Specificity

Injection of sample placebo (consisting of all matrix components with the exception of hederacoside C) in duplicate under the conditions of the developed HPLC method showed that there was no interference from sample matrix (Figure 2(b). In addition, peak purity testing of hederacoside C showed that the peak refers only to one component with peak-purity coefficient of 0.996. So the method was considered to be specific for hederacoside C analysis.

#### 3.2.5. Accuracy

This test was performed by the addition of known amounts of the hederacoside C standard to cough syrup placebo. The resulting mixtures, at three different concentration levels covering the method range, were assayed by the developed method analyzing 6 replicates at each concentration. The good recovery values obtained and their low RSD (less than 2%, Table 3) suggested that the accuracy of the proposed method was acceptable.

#### 3.2.6. Limits of Detection (LOD) and Quantitation (LOQ)

Based on the values of standard deviation of the response (SD), calculated as the standard deviation of *y*-intercepts of regression lines, and slope (S) obtained from the linear regression equation described above, the LOD and LOQ values were calculated, according to the ICH guidelines, as follows:

(1) $$\begin{array}{c} \text{LOD}=3.3*\left(\frac{\text{SD}}{\text{S}}\right),\\ \text{LOQ}=10*\left(\frac{\text{SD}}{\text{S}}\right). \end{array}$$

From these data, shown in Table 4, the developed method proved to be suitable for detection and quantitative determination of even very low drug contents (LOD = 0.011 mg/mL, and LOQ = 0.032 mg/mL). A validation of the calculated LOQ, as according to ICH guidelines, was also performed by the analysis, in duplicate, of 6 samples containing hederacoside C at the concentration of 0.03 mg/mL. The method proved to be with good repeatability (CV = 1.88) and intermediate precision (CV = 1.93) at the calculated LOQ. On the other hand, linearity and accuracy parameters were not tested because the calculated LOQ already occurs within the tested drug range (0.03–0.15 mg/mL) for these two parameters conducted as above.

## 4. Conclusions

A simple and reliable reversed phase high-performance liquid chromatographic method with UV detection for the quantification of hederacoside C, in the cough syrup has been developed and validated for its linearity, precision, specificity, and accuracy, providing a suitable and practical analytical method for the routine quality control analyses necessary for providing herbal medicines with high safety and efficacy.

## Figures and Tables

**Table 1:** Linear regression data of the HPLC method for the determination of hederacoside C in Ivy-Thyme cough syrup.

Parameter*	Value
Calibration range (mg/mL)	0.03–0.15
Regression equation slope	4612.8
*y*-Intercept	5000.3
Correlation coefficient (*r*)	0.9992

*Linear regression equation: *y* = 4612.8*x* + 5000.3.

**Table 2:** Repeatability and intermediate precision of the HPLC method for the determination of hederacoside C in Ivy-Thyme cough syrup.

Retention time mean (min ) ± SD *n* = 6	Concentration mean (mg/mL) *n* = 6	CV (%) (repeatability)	CV (%) (intermediate precision)
26.0 ± 0.1	0.1	1.83	1.81

*n*: Number of determinations (number of sample solutions (at 0.1 mg/mL) prepared and injected).

SD: Standard deviation.

CV: Coefficient of variation (RSD).

**Table 3:** Accuracy of the HPLC method for the determination of hederacoside C in Ivy-Thyme cough syrup placebo.

Level	% Recovery Mean (*n* = 6)	CV (%) *n* = 6
30%	100.45	1.81
100%	100.90	1.66
150%	99.69	0.67

CV: Coefficient of variation (RSD).

*n*: Number of determinations (number of sample solutions prepared and injected).

**Table 4:** Limits of detection (LOD) and quantitation (LOQ) of the HPLC method for the determination of hederacoside C in Ivy-Thyme cough syrup.

Standard deviation of the response (SD*)	LOD^a^ (mg/mL)	LOQ^a^ (mg/mL)
14831.8	0.011	0.032

*The value of SD was calculated as the standard deviation of *y* intercepts of regression lines plotted, at the chromatographic responses of the calibration solutions (0.03–0.15 mg/mL), parallel to the calibration curve (slope = 4612.8).

^a^For calculation of LOD and LOQ values, see Results and Discussion.
